# Snowball Effect of User Participation in Online Environmental Communities: Elaboration Likelihood under Social Influence

**DOI:** 10.3390/ijerph16173198

**Published:** 2019-09-01

**Authors:** Yali Zhang, Haixin Zhang, Zhaojun Yang, Jun Sun, Chrissie Diane Tan

**Affiliations:** 1 School of Management, Northwestern Polytechnical University, Xi’an 710072, China; 2 School of Economics and Management, Xidian University, Xi’an 710126, China; 3 College of Business and Entrepreneurship, University of Texas Rio Grande Valley, Edinburg, TX 78539, USA

**Keywords:** online environmental communities, social influence, perceived risk, perceived value, behavioral intention, actual participation

## Abstract

Ecological preservation and sustainable development depend on active public involvement. The emergence of online environmental communities greatly facilitates people’s participation in green endeavors. The population penetration of such platforms accelerates as existing users persuade people around them and media coverage further attracts public attention. This snowball effect plays an important role in the user base expansion, but the specific mechanism of social influence involved is yet to be examined. Based on the social influence theory, cognitive response theory, and elaboration likelihood model, this study establishes a research model depicting the relationship between persuasion in terms of social influence and outcomes in terms of behavioral intention and actual participation through the mediation of cognitive responses in terms of perceived value and perceived risk. Empirical results from survey observations show that social influence has both moderated (by education) and mediated (through perceived risk) effects on behavioral intention, which leads to actual participation. Meanwhile, social influence shapes the perceived value, which has a direct and strong impact on actual participation. These central and peripheral routes through which social influence affects individual participation yield useful theoretical and practical implications on human behavior with online environmental communities.

## 1. Introduction

Environment protection and sustainable development present both challenges and opportunities for all humanity. For this sake, online environmental communities have emerged as a social innovation that promotes public involvement in the value co-creation with ecological ventures [1,2]. Relying on the advantages of Web 2.0 technology and the diffusion of mobile social media, online environmental communities attract more people to pay attention to and participate in green activities and ecological projects with tangible and/or intangible contributions [3]. For instance, Ant Forest is a popular platform that encourages users to cultivate green habits to engage in low-carbon activities (e.g., walking and public transport) with “green energy” points, the accumulation of which leads to tree planting by ecological partners [4].

As a digital social innovation, online environmental communities change the way people participate in ecological activities. Population-wise, people’s participation rate with such platforms is growing fast as existing users persuade people around them to join them and the media coverage of the trend draws more public attention. Such a snowball effect plays an important role in attracting more individuals to online environmental communities. Nevertheless, it is not clear how the social influence involved leads to user engagement on the platforms. This study attempts to fill in the research gap by investigating the mechanisms through which social influence affects individual usage of online environmental communities. 

The remaining of this study is organized as follows. It first reviews relevant theoretical frameworks on the relationship between social influence and individual behavior. The discussion leads to the development of a research model depicting the direct and indirect effects of social influence on outcomes including behavioral intention and actual participation. Then, the methodology section describes a survey study to collect observations from online environmental community users. The results are presented, followed by the discussion of theoretical and practical implications.

## 2. Research Background

The social influence theory was proposed by social psychologist Kelman [5] and has been used in later frameworks to study individual behavior in social contexts, such as the theory of planned behavior (TPB) and technology acceptance model (TAM). The situation wherein the behavior of an individual is influenced by mass media and the people around can be defined as a social influence [6]. When users adopt new technologies, they are often influenced by the values and comments shared by the people around them [7]. Peers will affect an individual’s willingness to help others [8], and subjective norms will affect an individual’s willingness to participate in crowdfunding [9]. As a technology-enabled public endeavor, user engagement in online environmental communities is closely related to social influence, which mainly comprises media influence pertaining to the publicity of such platforms and peer influence concerning the word-of-mouth from important others.

The cognitive response theory posits a mechanism through which social influence affects individual behavior: In response to the persuasion from others, a person generates thoughts that shape subsequent psychological and observable actions [10]. In essence, cognitive responses serve as the mediators between social influence and behavioral outcomes. Thus, the theory provides a useful lens to examine how the people around influence an individual’s decision to use an online environmental community. Yet, the cognitive response theory just provides a general framework, and researchers still need to specify exact cognitive responses under each context. Based on the information from mass media, social media, and word-of-mouth describing people’s good or bad experiences with online environment communities, a potential user can have positive or negative cognitive responses. Correspondingly, they are identified as perceived value and perceived risk in this study.

Rooted in the consumer behavior field, perceived value refers to the subjective evaluation of whether the product or service meets customer needs after purchase and usage [11]. It can be divided into perceived price value, perceived quality value, perceived emotional value, and perceived social value [12]. With the fierce competition and high degree of homogeneity in today’s markets, the impacts of the perceived emotional value and perceived social value on purchase intention become more important albeit the traditional influences of the perceived price value and perceived quality value on consumers’ purchase intention [13]. Similarly, the perceived emotional value and perceived social value also affects the donation intention in crowdfunding projects in public welfare [14]. Since online environmental communities do not concern physical commodities, this study focuses on its perceived emotional value and perceived social value to users. The perceived emotional value refers to the sum of the emotions and feelings an individual experiences from participating in online environmental communities. The perceived social value refers to the utility of social benefits that an individual perceives from participating in online environmental communities. 

Contrary to the perceived value, perceived risk refers to consumers’ perception of the uncertainty of consumption results that may make them unhappy [15]. The dimensions of the perceived risk include the financial risk, physical risk, functional risk, social risk, psychological risk, and time risk [16]. In the Web 2.0 era, it is necessary to take the perceived privacy risk into account as an important dimension of the perceived risk in the virtual world [17,18]. As online environmental communities do not involve monetary transactions, this study measures the perceived risk of participants from two dimensions: Perceived privacy risk and perceived operational risk. The perceived privacy risk refers to the possibility perceived by an individual that the participation in an online environmental community leads to the tracking and disclosure of personal information (e.g., habits). The perceived operational risk refers to the potential losses (e.g., points, contacts) that a participant’s own operational mistakes may incur in the use of an online environmental community.

The elaboration likelihood model (ELM) identifies dual processes from persuasion to behavioral change in terms of central and peripheral routes of information processing [19]. Through the central route, an individual evaluates the messages received from positive and negative aspects and decides whether to take action. When strong cues are present supporting or rejecting the credibility of an information source, however, a person may skip the evaluation and decision-making process but take the peripheral route to simply accept or decline the persuasion. In the context of online environmental communities, the trustworthiness of word-of-mouth from people known to a potential user represents social cues. When a close friend shares the experiences with such a platform with enthusiasm, for instance, social cues can be strong enough for an individual to accept the belief and give it a try. 

## 3. Research Model

**Figure 1:**
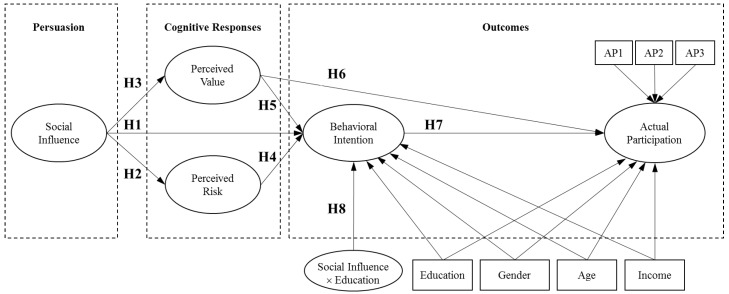
Research Model. H = Hypothesis. AP = Actual Participation.

Based on the aforementioned theoretical frameworks, this study develops a research model as shown in Figure 1. The cognitive response theory serves as a general framework connecting the major components of persuasion, cognitive responses, and outcomes. In addition to the social influence theory pertaining to persuasion, the ELM helps identify the roles that different cognitive responses play in affecting the outcomes. To accommodate both central and peripheral routes, both psychological and observable outcomes are included in terms of behavioral intention and actual participation, respectively. The theory of reasoned action posits that behavioral intention is formed on the basis of cognitive evaluation and reasoning [6]. Thus, the mediated relationship between the cognitive response and actual participation through behavioral intention indicates a central route, and the direct relationship indicates a peripheral route. 

It is a common practice to include both behavioral intention and actual behavior in psychology-based studies, though the latter is merely included as a predicted variable of the former [20,21]. For instance, Shneor and Munim [9] studied individuals’ participation in corporate crowdfunding and found that subjective norms positively affected behavioral intention that leads to actual participation subsequently. From the perspective of ELM, however, a cognitive response may directly lead to actual behavior, in addition to behavioral intention as a mediator. In this study, behavioral intention captures the subjective disposition of an individual to participate in an online environmental community. As the eventual outcome variable, actual participation is a formative construct comprising three stages of online environmental community engagement in terms of opportunity exploration, activity contribution, and status tracking. Whereas behavioral intention and other reflective constructs in the model capture psychological states, the formative construct indicates observable behavior. The inclusion of both reflective and formative constructs makes the model more meaningful by addressing the “so what” question beyond psychological processes.

Researchers often include demographic variables as control variables to make the estimation of the main effects more accurate. For instance, Im, et al. [22] found that the user age and income negatively and positively correlate with the consumer adoption of new products, respectively. Martin, et al. [23] found that age and education are covariates with the user adoption of telecommunication innovation. Other studies have shown that gender makes a difference in the user attitude toward computers, wherein men are less anxious about computers than women [24]. For more accurate estimates of main relationships, therefore, this study control for the effects of demographic variables, including education, gender, age, and income, on both outcomes of behavioral intention and actual participation.

Social influence impacts human behavior through cognitive processes as well as social construction [25]. According to the social identity theory, people will classify themselves into certain social categories [26]. Participating in an online environmental community gives its users a sense of common purpose and shared identity. In the information systems (IS) literature, the behavioral intention to use a system is susceptible to social influence in the form of subjective norms [7]. If people around are actively using a system, an individual is likely to adopt it as well to get assimilated to the group. In the context of online environmental communities, therefore, people’s intention to participate is largely shaped by such normative beliefs [27]. Engaging in ecological activities would make a person look like others who are environmentally aware and active. Hence, the following research hypothesis.

**Hypothesis** **1.** *Social influence positively affects behavioral intention.*

In addition to its direct impact on behavioral intention, social influence may have indirect effects through the mediation of cognitive responses. That is, social influence affects an individual’s perceptions of the risk and value associated with an online environmental community, which then shape the intention to use the platform. On the risk side, researchers found that social influence affects an individual’s evaluation of uncertainties and potential harms [28]. It is found that recommendations from relatives and friends, brand reputation, and positive comments reduce consumers’ perception of risk [29,30]. Through a conformity effect, individuals in social networks initially differ in risk perceptions, but over time become more alike under the mutual influence [31]. On digital platforms, the positive electronic word-of-mouth serves as a risk mitigation mechanism [32].

**Hypothesis** **2.** *Social influence negatively affects perceived risk.*

Similarly, social influence has an impact on perceived value. Opinion leaders’ input [33] and online comments [34] can change consumers’ perceived value of a product. The word-of-mouth from close friends, relatives, and colleagues regarding the ecological impacts that they make through online environmental communities will shape an individual’s perception of how beneficial it is to use such a platform. In addition to the interpersonal communication and social network interaction, the traditional mass media also play an important role in shaping people’s value beliefs on issues of public interests through their media dependency [35].

**Hypothesis** **3.** *Social influence positively affects perceived value.*

As a behavioral inhibitor, perceived risk is known to negatively affect behavioral intention [36]. To participate in online environmental communities, users are often required to provide personal information, leading to the risk of privacy leakage [37]. In addition, participation may potentially disrupt or even intrude people’s personal lives, as pro-environment activities take time and effort [38]. Based on the evaluation of all risks, people may become hesitant to participate in online environmental communities.

**Hypothesis** **4.** *Perceived risk negatively affects behavioral intention.*

In contrast to the perceived risk, perceived value is found as a positive predictor of behavioral intention [11,39]. In the context of this study, when people have a stronger belief that their engagement in online environmental communities will contribute to a greener world, they are more willing to use such platforms. Such rational reasoning is based on the central route of information processing when the person has to evaluate the messages from various sources. When people are exposed to different online environmental communities through mass media, for instance, they may compare their values to select which one to use.

**Hypothesis** **5.** *Perceived value positively affects behavioral intention.*

In addition, researchers notice that perceived value sometimes directly brings about actual behavior [40,41]. From the perspective of ELM, this is explainable: People are likely to take the peripheral route of information processing when persuasion is from trustworthy sources. When a close friend recommends an online environmental community, for example, a person’s perceived value may bypass behavioral intention but directly lead to actual participation.

**Hypothesis** **6.** *Perceived value positively affects actual participation.*

IS researchers have found empirical evidence on the relationship between behavioral intention and actual usage of organizational systems [42] as well as individual systems like micro-blogs [43] and electronic commerce [44]. In the Web 2.0 and mobile computing era, behavioral intention can still predict actual usage that is ubiquitous and collaborative in nature, such as mobile payment and social networking [45,46]. The same would stand for online environmental community engagement.

**Hypothesis** **7.** *Behavioral intention positively affects actual participation.*

As an indicator of socio-economic status, education is likely to interact with social influence in affecting individual behavior [47]. The more educated a person is, the more rational and independent the individual becomes, and the less likely he or she is to be influenced by others. As for the participation in an online environmental community, a less educated individual is more likely to be persuaded by others. In addition to its direct impact as controlled for in this study, therefore, education is modeled as a negative moderator.

**Hypothesis** **8.** *Education negatively moderates the relationship between social influence and behavioral intention.*

## 4. Methodology

To test the research model, survey observations were collected with online questionnaires. Most of the measurement items are adapted from the existing studies. The scale of social influence is based on Venkatesh, Morris, Davis and Davis [7]. The perceived value is measured with items adapted from Sweeney and Soutar [12,48]. The perceived risk and behavioral intention are captured with the instruments adapted from Featherman and Pavlou [49] and Stone and Gronhaug [48]. The scale of actual participation is self-developed comprising three items corresponding to the opportunity exploration, activity contribution, and status tracking stages of participation in online environmental communities. Listed in the Appendix A, all the items use a five-point Likert scale from “strongly disagree” to “strongly agree.” 

The target population comprises users of online environmental communities. As the biggest developing country that faces the challenge of balancing ecological conservation and economic development, China saw quite a few such platforms established in recent years, exemplified by Ant Forest, Rice Welfare, and Green Future. They attract millions of people participating in various crowdfunding activities with tangible and intangible contributions to ecological endeavors. Survey responses were collected from online environmental community users in China through the questionnaire website “Questionnaire Star” [50] for a period of one month. In addition to the measurement items, the questionnaire asked each participant to check all the online environmental communities he or she had used. The survey links were sent along with the invitations to the user circles on main social media including WeChat, QQ, Weibo, and Alipay. 

Altogether, 312 responses were collected, but 18 of them did not indicate any online environmental communities used. Thus, there were 294 valid observations, resulting in an effective response rate of 94.2%. Out of the 294 participants in the final sample, 267 (90.8%) had used Ant Forest, which shares Alipay’s user base of over 900 million [51]. Due to its publicity, Ant Forest is known to almost everyone in China who participated in online environmental communities. The high percentage of its users in the sample confirms the representativeness of the data source. There were 73 (24.8%) participants who had used Rice Welfare, which is an innovative mobile Internet platform established in December 2012. Through practicing greener and healthier lifestyles (e.g., walking), users can accumulate virtual rice grains to support public welfare projects. Having about the same number of users 70 (23.8%), Green Future is an online environmental community jointly established by the China Environmental Culture Promotion Association and Shanghai Automotive Industry Corporation (SAIC) General Motors Corporation Limited on 5 June 2015. Only eight participants (2.7%) have used other platforms. As shown in Table 1, the participants that had an almost equal gender mix, are relatively young and well-educated. Young people generally have a higher educational level and are more willing to pay attention to and accept new things. The distributions of occupations and income levels are also in line with the general population. 

The common method bias (CMB) was assessed with Harman’s one-factor test. The results of the exploratory factor analysis revealed that less than 50% of the common variance was explained by the first principle component, indicating no serious CMB. In addition, alleviating the CMB concern is the fact that the research variables used in this study are of different natures. Unlike the other reflective latent constructs in the research model, the final outcome variable—actual participation—is a formative latent construct. To test the model, this study conducts structural equation modeling (SEM) based on the partial least squares (PLS), as the PLS-based SEM is more capable of handling formative constructs than the traditional covariance-based SEM [52]. 

## 5. Results

Table 2 shows the results of measurement validation for reflective constructs in the research model. The response patterns were consistent with the expectations, as the mean score of the perceived risk was negative, whereas the others were positive, and standard deviations (SD) indicated reasonable dispersions. All the values of composite reliability (CR) were above 0.7, suggesting that the reliability of responses is acceptable. Convergent validity was supported given that the average variance extracted (AVE) of each variable was greater than 0.5. There was evidence for discriminant validity as well since the largest correlation coefficient (i.e., 0.737) was lower than the smallest square root of AVE (i.e., 0.755). Regarding the formative construct of actual participation, the variance inflation factor (VIF) values of three indicators were below the threshold of five (1.106, 1.107, 1.007, respectively for AP1, AP2, AP3), supporting that they capture distinct dimensions of opportunity exploration, activity contribution, and status tracking. The multi-collinearity among the control variables was not excessive, as their correlation coefficients were all beneath 0.5 (the highest was 0.497 between Income and Age, followed by 0.230 between Income and Education) and VIFs were way below the threshold of five (the highest was 1.405).

**Figure 2:**
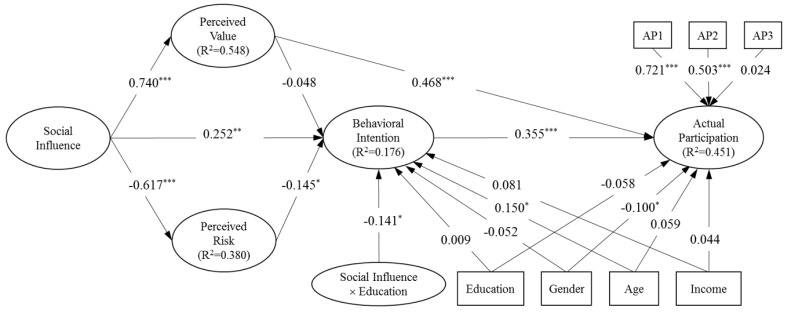
Standardized model estimates. *** *p* < 0.001. ** *p* < 0.01. * *p* < 0.05. two-tailed test.

Figure 2 shows the estimated model. The overall model fit was acceptable as the standardized root mean square residual (SRMR) was 0.077, less than the threshold of 0.10 [53,54]. In addition, the model’s explanatory power for each endogenous variable is indicated by the associated coefficient of determination (i.e., *R*^2^). Social influence was able to explain over half of the variance (54.8%) in perceived value and over one third (38.0%) in perceived risk. As for the outcome variables, more variance in actual participation (45.1%) was explained than that in behavioral intention (17.6%), contrary to the pattern found in social psychology and other fields (i.e., the *R*^2^ of behavioral intention is usually much larger than that of actual participation). 

Regression path estimates indicated that perceived value is a stronger predictor on actual participation than behavioral intention (i.e., 0.468 compared to 0.355). Meanwhile, perceived value did not have a significant impact on behavioral intention, which is the only hypothesized relationship (Hypothesis 5) not supported. The other two predictors of behavioral intention, social influence, and perceived risk, exhibited moderate influences (0.252 with *p*-value < 0.01 level and −0.145 with *p*-value < 0.05, respectively). In contrast, social influence had much bigger impacts on perceived value and perceived risk (0.740 and −0.617, both significant at the 0.001 level). The mediated relationship between social influence and actual participation through perceived value (i.e., 0.740 × 0.468 = 0.346) was much stronger than that through behavioral intention (i.e., (0.252 + (−0.617 × −0.145)) × 0.355 = 0.121).

Among the control variables, age had a positive effect on behavioral intention, and gender (male = 1, female = 0) had a negative effect on actual participation, whereas education and income did not make too much a difference in either. The results also suggest that the user behavior in online environmental communities is somewhat distinct from the typical IS user behavior, in which age and gender exhibit opposite effects, whereas education and income make differences. 

**Figure 3:**
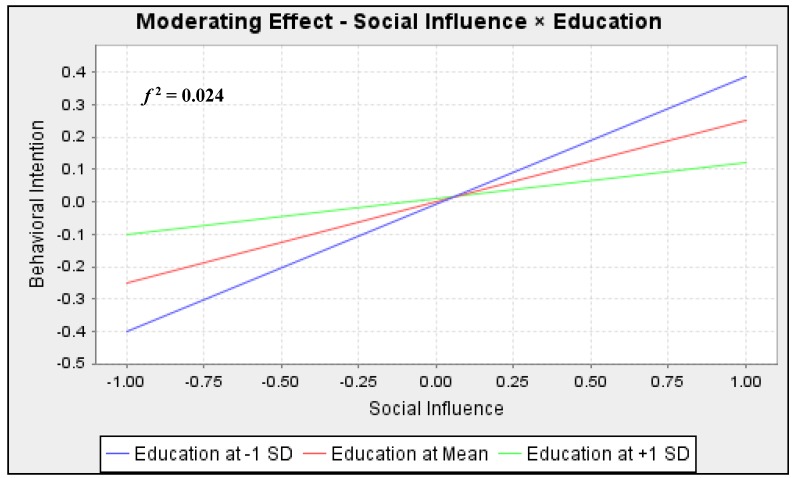
Moderating effect of education.

As expected, however, education negatively moderated the effect of social influence on behavioral intention (Hypothesis 8). Figure 3 shows that the slope between social influence and behavioral intention decreases at higher education levels. and the *f*-square value of 0.024 suggests a relatively large moderation effect size [52]. This confirms that better-educated individuals are less likely to be influenced by others but rather more independent in decision-making regarding whether or not to participate in online environmental communities.

## 6. Discussions 

Unlike most of the existing studies on online environmental communities that focus on the characteristics of technological platforms [55,56], this study examines the mechanisms through which social influence, perceived value, and perceived risk affect both behavioral intention and actual participation. The results provide supporting evidence to seven out of the eight research hypotheses. This confirms the validity of the overall research model developed on the basis of the social influence theory, cognitive response theory, and elaboration likelihood model (ELM). User engagement in online environmental communities is indeed a technology-mediated individual behavior embedded in a social context. 

Social influence largely shapes the perceived value, a cognitive response that had a direct impact on actual participation. Meanwhile, the rationality of such behavior is captured with the behavioral intention that is shaped by the normative belief and risk evaluation, respectively. A positive social influence not only facilitates decision-making but also mitigates the perceived risk, suppressing its role of a behavioral inhibitor. Nevertheless, the effect of social influence on behavioral intention is offset by education. It is found that the model is able to explain more variance in actual participation than behavioral intention, suggesting that people’s participation in online environmental communities is distinct from most rationality-based social behavior in the literature.

The results provide an insight into the mechanism of how social influence affects actual participation in online environmental communities. It is an individual choice to join an online environmental community, but user participation is collective in nature. That is, individual participations add up to a social trend that influences future participation. For existing users, such a loop leads to reinforced usage as well as peer-to-peer recommendation. When such social cues are present, people may bypass the central route of information processing based on the evaluation of pros and cons, but take the peripheral route that directly leads to actions. In this study, three-fourths of social influence’s total effect was carried from perceived value directly to actual participation (i.e., 0.346/(0.346 + 0.121) = 74.09%), only the rest one fourth through the mediation of behavioral intention. Thus, the social influence took effect mainly through the peripheral route rather than the central route, suggesting that peer influence is more effective in promoting online environmental community participation than media influence.

The strong impacts of social influence on user participation in online environmental communities also explain the seemingly contradictory findings regarding the effects of control variables. In this study, age had a positive effect on behavioral intention, as social influence is stronger when the exposure is longer in period and broader in scope. On the other hand, gender had a negative effect on actual participation: Though males are generally more open to innovation, females are more social. As major indicators of socio-economic status, income, and education are moderately correlated with each other, and their positive effect on technology adoption are offset by education’s mitigation of social influence.

This study has limitations, which provide directions for future research. In particular, the results are obtained with the observations collected from one country. Though China is the world’s factory while having the largest Internet population, the single-country sample limits the generalizability of findings to other countries and regions. For this sake, multi-country studies can be carried out as environmental protection is a global issue that everyone needs to pay attention to. This also enables cross-cultural analyses to examine the influence of national cultures on people’s participation in online environmental communities.

## 7. Conclusions

Integrating the social influence theory, cognitive response theory, and elaboration likelihood model (ELM), this study proposes a research model on the antecedents of user participation in online environmental communities. Empirical results support most of the hypothesized relationships and reveal the mechanisms through which social influence affects actual participation. The primary route is through the mediation of perceived value, and the secondary route is through the mediation of behavioral intention. Education acts as an insulation layer between social influence and behavioral intention, whereas there is a bypass route through perceived risk. The insights from integrating multiple theoretical frameworks contribute to the understanding of how social influence affects individual behavior on digital public platforms.

The findings yield important theoretical implications. First, they reveal the mechanisms through which social influence affects people’s online environmental community participation. The cognitive response theory helps identify perceived value and perceive risk as the mediators between persuasion and outcomes. Yet they may carry the effect of social influence in different manners onto two outcomes, behavioral intention as the intermediate one and actual participation as the eventual. The results suggest two parallel paths from social influence to actual participation: One through perceived value and the other through behavioral intention. From the perspective of ELM, they represent peripheral and central routes of information process, respectively. Bypassing behavioral intention, the peripheral route is found dominant in this study, suggesting that peer influence is the main force promoting online environmental community participation with social cues. Though less prominent, the significance of the central route through behavioral intention suggests that media influence still makes a difference through publicity. Rooted in user participation, the social influence in both routes yields a snowball effect on the continuous expansion of the user base, albeit a more direct push from peers than media. 

Consequently, the different roles that the perceived value and perceived risk play in online environmental community user behavior extend the cognitive response theory. By including both psychological and observable outcomes, it is possible to empirically distinguish the mechanisms concerning different cognitive responses. The result that education negatively moderates the relationship between social influence and behavioral intention indicates that people of relatively high education tend to think independently. This confirms the validity of distinguishing central and peripheral routes based on whether behavioral intention is included as a mediator or not. Though both are cognitive responses to social influence, perceived value directly leads to actual participation, and perceived risk shapes behavioral intention first. A cognitive response, therefore, may be involved in the central or peripheral route depending on the source of persuasion. 

In particular, the peripheral route involving perceived value identified in this study extends the literature on perceived value. The results show that perceived value may serve as the direct link from social influence to observable behavior. This mechanism becomes obvious especially when the information source is close and trustworthy. On the other hand, perceived risk plays a more traditional role in the central route due to the cognitive evaluation involved. Nevertheless, the finding that the publicity of a social platform on mass media is likely to mitigate the perceived risk contributes to the research on perceived risk. In theory development, therefore, behavioral intention can be modeled as the full mediator between perceived risk and actual behavior, but as the partial mediator for perceived value. Depending on the result indicating full mediation or no mediation, the perceived value can be a part of the central or peripheral route, respectively (in the case of partial mediation, central and peripheral routes are indistinguishable).

The findings yield some helpful practical implications as well. For the individual participants of online environmental communities, they should form groups in the virtual world to exchange experiences and encourage each other. That will optimize the social influence and promote the continuous engagement that is critical for the success of online social welfare. For the organizers of online environmental communities, it is critical to provide trustworthy information and implement security measures to help participants increase perceived value and reduce perceived risk. In particular, they need to provide timely feedback of the welfare project progress and outcome to deepen the user involvement further, as status tracking was the only dimension that was not found significant among the formative indicators of actual participation. When users are informed of the impacts that they have made, they are more likely to see the value of online environmental communities and tell others about their experiences.

## Figures and Tables

**Table 1:** Participant Profile (*n* = 294).

Feature	Options	Frequency (%)
Gender	Male (coded as 1)	149 (50.68%)
	Female (coded as 0)	145 (49.32%)
Age	Below 18	13 (4.42%)
	18–25	124 (42.18%)
	26–35	108 (36.73%)
	36–45	35 (11.9%)
	46–55	9 (3.07%)
	Over 56	5 (1.7%)
Occupation	Student	96 (32.66%)
	Government employee	25 (8.5%)
	State-owned enterprise employee	57 (19.39%)
	Private enterprises employee	80 (27.21%)
	Freelancer	30 (10.2%)
	Other	6 (2.04%)
Educational level	Associate degree or below	42 (14.28%)
	Bachelor degree	121 (41.16%)
	Master’s degree	117 (39.8%)
	Doctorate degree	14 (4.76%)
Monthly Income	Below CNY 2000	87 (29.59%)
	CNY 2001–4000	54 (18.37%)
	CNY 4001–6000	68 (23.13%)
	CNY 6001–8000	55 (18.71%)
	Above CNY 8001	30 (10.2%)

**Table 2:** Measurement validation.

Variable	Mean (SD)	CR	AVE	1	2	3	4
Social Influence	4.16 (0.61)	0.817	0.598	**0.773**			
Perceived Value	4.18 (0.62)	0.841	0.570	0.737	**0.755**		
Perceived Risk	1.77 (0.76)	0.895	0.630	−0.614	−0.596	**0.794**	
Behavioral Intention	4.03 (0.65)	0.848	0.582	0.318	0.232	−0.269	**0.763**

Note: The bolded values on the diagonal of the correlation matrix are the square roots of the average variance extracted (AVE). All correlation coefficients were significance at the 0.01 level. CR = composite reliability. SD = standard deviations.

## Appendix A

### Measurement Items

Social Influence

1. Mass media are promoting online environmental communities.
2. People important to me think that I should participate in an online environmental community.
3. Participating in an online environmental community can help me integrate into the current trend.

Perceived Value

1. Participation in an online environmental community brings me a sense of pride.
2. Participation in an online environmental community brings me happiness.
3. Participation in an online environmental community helps me make a good impression on others.
4. Participation in an online environmental community helps me get praise from others.

Perceived Risk

1. When I participate in an online environmental community, Internet hackers may control my account.
2. My information entered to an online environmental community may be used by others without my knowledge.
3. When I participate in an online environmental community, my habits may be leaked or tracked.
4. I am worried that my operational mistakes in an online environmental community will incur some loss.
5. I am afraid that the damages caused by my operational mistakes will be unrecoverable.

Behavioral Intention

1. I want to participate in an online environmental community.
2. I will engage in an online environmental community in the future.
3. I will continue to pay attention to online environmental communities.
4. I will recommend others to participate in online environmental communities.

Actual Participation

AP1. I frequently look for opportunities in an online environmental community.
AP2. I contribute to the activities in an online environmental community on a regular basis.
AP3. I track the status of my involvement in an online environmental community.
